# MicroRNA-141 inhibits migration of gastric cancer by targeting zinc finger E-box-binding homeobox 2

**DOI:** 10.3892/mmr.2015.3789

**Published:** 2015-05-15

**Authors:** YING DU, LINGFEI WANG, HONGHAI WU, YIYIN ZHANG, KAN WANG, DINGTING WU

**Affiliations:** 1 Department of Endocrinology, Sir Run Run Shaw Hospital, School of Medicine, Zhejiang University, Hangzhou, Zhejiang 310016, P.R. China; 2 Department of Pharmacy, Second Affiliated Hospital, School of Medicine, Zhejiang University, Hangzhou, Zhejiang 310009, P.R. China; 3 Institute of Pharmacology and Toxicology, Zhejiang University, Hangzhou, Zhejiang 310058, P.R. China; 4 Department of Gastroenterology, Sir Run Run Shaw Hospital, School of Medicine, Zhejiang University, Hangzhou, Zhejiang 310016, P.R. China

**Keywords:** gastric cancer, migration, zinc finger E-box-binding homeobox 2, microRNA-141

## Abstract

Human microRNA (miR)-141 is a member of the miR-200 family, which has been reported to be downregulated in gastric cancer, and involved in the proliferation of gastric cancer cells. However, little is currently known regarding its role in the migration of gastric cancer. The present study investigated the function of miR-141 in gastric cancer cell migration, and evaluated the contribution of zinc finger E-box-binding homeobox 1 and 2 (ZEB1/2) in miR-141 mediated migration of gastric cancer cells. The expression levels of miR-141 and its potential ZEB1/2 targets were examined by quantitative polymerase chain reaction (qPCR) and western blotting, respectively. The migration of SGC-7901 and HGC-27 gastric cancer cells, which had been transfected with an miRNA precursor, was examined by cell migration and wound healing assays. A luciferase activity assay was used to validate whether ZEB1/2 was a direct target of miR-141. The results demonstrated that overexpression of miR-141 markedly inhibited the migration of gastric cancer cells *in vitro*. Forced overexpression of miR-141 significantly reduced the luciferase activity of the 3′-untranslated region of ZEB2 in gastric cancer cells. Furthermore, the mRNA and protein expression levels of ZEB2 were reduced in cells overexpressing miR-141, whereas the protein expression levels of E-cadherin were increased. In gastric tumor samples the expression levels of ZEB2 were inversely correlated with the expression of miR-141. These results suggest that miR-141 may be involved in the inhibition of gastric cancer cell migration, and that ZEB2 is a target gene of miR-141.

## Introduction

Gastric cancer is the fourth most common type of cancer and the second most frequent cause of cancer-associated mortality worldwide (1). The global age-standardized mortality rates are 14.3 per 100,000 in men, and 6.9 per 100,000 in women (2). In 2013, the estimated number of new cases of stomach cancer in the United States reached 21,600, with a mortality rate of ~10,990 (3). Despite recent advances in the diagnosis and treatment of gastric cancer, the majority of patients are diagnosed with advanced gastric cancer, and the survival rate remains unsatisfactory. Invasion and metastasis are the hallmarks of advanced gastric cancer progression; therefore, understanding the pathogenesis of gastric cancer metastasis is critical to improve the survival rate of patients.

Micro (mi)RNAs are a class of short, non-coding RNAs which have been reported to be involved in numerous physiological processes, and to have important roles in diseases such as cancer (4). Each miRNA can target numerous genes, either by degradation of mRNAs or by inhibition of translation. In cancer, miRNAs have been identified to target oncogenes or tumor suppressors, and therefore may positively or negatively regulate cancer cell proliferation, migration, and invasion (4,5). In gastric adenocarcinoma, overexpression of miR-181a has been shown to increase cell proliferation and to inhibit apoptosis by reducing the expression of tumor suppressor Krueppel-like factor 6 (6). In addition, miR-375 is downregulated in gastric cancer and its overexpression inhibits cancer cell proliferation by targeting the oncogene Janus kinase 2 (7).

miR-141 is a member of the miR-200 family, which has been shown to be downregulated in gastric tumor tissue. In addition, overexpression of miR-141 significantly reduces the proliferation of gastric cancer cells (8). Notably, it has been demonstrated that miR-141 may inhibit cell migration and invasion in colorectal, pancreatic, and hepatocellular carcinoma (9–11). Therefore, the present study hypothesized that miR-141 may also have a role in the migration of gastric cancer cells.

Epithelial to mesenchymal transition (EMT) is a fundamental biological disease process, during which polarized immotile epithelial cells are converted to motile mesenchymal cells. EMT can lead to tumor invasion and metastasis (12,13). The zinc finger E-box-binding homeobox, including ZEB1 and ZEB2, inhibits the translation of E-cadherin, the loss of which is a marker of EMT. The miR-200 family has previously been reported to suppress EMT through translational inhibition of ZEB mRNA in various types of cancer (14). miR-141 has been shown to exclusively target ZEBs in renal, colorectal, and head and neck squamous carcinoma cells (9,15,16). Therefore, the present study aimed to determine whether miR-141 targets ZEB in gastric cancer, and whether it regulates the migration of cancer cells.

## Materials and methods

### Clinical samples and cell lines

A total of 18 gastric mucosa samples, including 9 non-malignant mucosa and 9 malignant mucosa samples, were obtained by gastroscopy from the Sir Run Run Shaw Hospital, Zhejiang University School of Medicine (Hangzhou, China). Written informed consent was obtained from the patients prior to collection, and the study was approved by the Ethics Committee of the Sir Run Run Shaw Hospital School of Medicine Zhejiang University. Patient information is shown in Table I. All of the samples were immediately frozen and stored in liquid nitrogen for RNA analysis. Histopathological investigation was performed by two professional pathologists independently. Other clinical data were obtained from medical records within the Sir Run Run Shaw Hospital. The SGC-7901 gastric cancer cells (Shanghai Institutes for Biological Sciences, Shanghai, China) were cultured in RPMI 1640 medium (Invitrogen Life Technologies, Carlsbad, CA, USA). The HGC-27 human gastric cancer cells (Shanghai Institutes for Biological Sciences) were maintained in Dulbecco's modified Eagle's medium (Invitrogen Life Technologies). All media were supplemented with 10% fetal bovine serum (Gibco-BRL, Gaithersburg, MD, USA). Cells were maintained in a humidified atmosphere with 5% CO_2_ at 37°C.

### Construction of plasmids

The putative miR-141-binding sites in the ZEB2 3′-untranslated region (UTR) region were detected using Targetscan software (http://www.targetscan.org). To produce the pMIR-ZEB2 plasmid, the human ZEB2 3′-UTR was amplified by polymerase chain reaction (PCR) using the following primers: Forward 5′-ATG TGC TCG CAC TAC AATGC-3′, and reverse 5′-ATT GGT ACC AGT CAA AAT TAT TGC-3′ (GenePharma Co., Ltd., Shanghai, China) prior to being cloned into a pMIR-REPORT vector (Ambion Life Technologies, Carlsbad, CA, USA). The mutation on the miR-141-binding sites in human ZBE2 3′-UTR was generated by overlap PCR as described previously (17). The primer sequences were as follows: Forward 5′-AGCTT CATTC TTGAG CTCACC-3′ and reverse 5′-GGTGAG CTCAA GAATG AAGCT-3′. All constructs were confirmed by sequencing.

### RNA extraction and reverse transcription-quantitative (RT-q)PCR

Tissue samples were homogenized manually in liquid nitrogen then immediately transferred to TRIzol reagent (Ambion Life Technologies). The total RNA was isolated from the tissue samples and cell lines using the mirVana miRNA Isolation kit (Ambion Life Technologies). Mature miRNAs were reverse transcribed using the TaqMan^®^ MicroRNA reverse transcription kit (Applied Biosystems Life Technologies, Foster City, CA, USA), and qPCR was performed using TaqMan microRNA assays with specific primers for hsa-miR-141 (P/N, 4373137; Applied Biosystems Life Technologies). qPCR was performed on the Applied Biosystems 7500 Real-Time PCR systems (Applied Biosystems). The cycling conditions were as follows: 95°C for 30 sec, annealing at 60°C for 30 sec then 72°C for 30 sec for 30 cycles then maintained at 4°C until further analysis. Comparative qPCR was performed in triplicate, including non-template controls. All data was normalized to U6 expression (P/N, 4373381, Applied Biosystems). Fold change was determined as 2^−ΔΔCt^ and miR-141 expression in endoscopic samples was also normalized to U6 expression using the 2^−ΔΔCt^ method (18).

### Cell transfection

To determine the effects of miR-141 on cell proliferation, 5×10^3^ SGC-7901 and HGC-27 cells were transfected with either a 25 nM Pre-miR™ miR-141 precursor molecule (miR-141; Ambion Life Technologies), or a negative control #1 Pre-miR™ miRNA precursor molecule (Negative; Ambion Life Technologies), using siPORT™ Amine Transfection Agent (Ambion Life Technologies) in a 96-well plate. The expression of miR-141 was detected by qPCR 24 h post-transfection. An MTT assay was performed at 24 h post-transfection. Cells were seeded into 96-well plates containing a final volume of 100 *µ*l/well and treated with the miRNA as indicated. Subsequently, 10 *µ*l MTT solution (Bio Basic Inc., Amherst, NY, USA) was added per well to achieve a final concentration of 0.45 mg/ml. Following incubation for 2 h at 37°C, 100 *µ*l solubilization solution was added to each well to dissolve formazan crystals. The absorbance of the samples was measured using a spectrophotometer reader (Multiskan FC 51119000, Thermo Fisher Scientific, Inc., Waltham, MA, USA). at 490 nm. Each assay was performed in triplicate and repeated three times.

### Cell migration assay

A Boyden chamber system (Costar, Corning, Inc., Tewkesbury, MA, US) was used for the transwell migration assay. The SGC-7901 and HGC-27 cells (5×10^4^) were transfected with either 25 nM miR-141 or negative control in 24-well plates for 24 h. The cells were then trypsinized and 10^4^ cells were seeded into each insert in culture medium, and the same medium was placed in the well below. Following a 24 h incubation period, the cells remaining in the top inserts were removed using a cotton swab, and the cells that had migrated through the filter were fixed with 75% ethanol for 30 min, followed by 0.1% crystal violet staining (Sigma-Aldrich, St. Louis, MO, USA) for 20 min. The ability of the cells to migrate to the lower chamber was visualized, and the images were captured using an inverted microscope (Leica DMI 4000B, Leica Microsystems, Wetzlar, Germany).

### Wound healing assay

Approximately 5×10^4^ cells were seeded in 24-well plates and cultured until 70–80% confluent. The cells were transfected with miR-141 and controls as previously described. Wounds were established using a p20 pipet tip and the cells were allowed 24 h to migrate into the wounds. To assess the migration of the cells across the artificial wound, a total of five optical fields (magnification, ×10) were randomly selected and analyzed using a Leica DMI 4000B inverted microscope with the Leica application suite software (Leica Microsystems).

### Western blot analysis

Protein samples were extracted using the Total protein extraction kit (Beyotime Institute of Biotechnology, Shanghai, China). Protein concentrations were quantified using a Lowry protein assay kit (Majorbio Biotech Co., Ltd., Shanghai, China). Protein samples were size-fractionated by 10% SDS-PAGE using 10 *µ*g protein and then transferred to polyvinylidene difluoride membranes (EMD Millipore, Bedford, MA, USA). The blots were blocked for 1 h in 5% milk/Tris-buffered saline-0.1% Tween 20 (TBS-T), and then incubated with primary antibodies at 4°C overnight. The blots were then washed three times for 15 min with TBS-T, followed by incubation with the secondary antibodies in 5% milk/TBS-T for 1 h, and then washed three times for 15 min with TBS-T. The membranes were briefly incubated with enhanced chemiluminescence detection reagent (GE Healthcare, Pittsburgh, PA, USA) to visualize the proteins, and then exposed in a cassette to an X-ray film (Carestream Health, Inc, Shanghai, China) for several minutes. The blots were quantified using Quantity One 1-D analysis software, version 4.62 (Bio-Rad Laboratories, Inc., Hercules, CA, USA).

The primary antibodies used were as follows: Anti-E-cadherin (cat no. 610405), purchased from BD Biosciences (Franklin Lakes, NJ, USA), and anti-β-actin (cat no. sc-47778), anti-ZEB1 (cat no. sc-25388) and anti-ZEB2 (cat no. sc-48789), which were purchased from Santa Cruz Biotechnology, Inc. (Dallas, TX, USA). The secondary antibodies used were as follows: Goat anti-mouse IgG HRP (cat. no. 2A-10004302-1), goat anti-rabbit IgG HRP (cat. no. 2A-10004301-1) (Cayman Chemical, Ann Arbor, MI, USA) and rabbit anti-goat IgG HRP (cat. no. SC-2768; Santa Cruz Biotechnology Inc.).

### Luciferase activity assay

A total of 4×10^4^ cells were seeded in 24-well plates 24 h prior to transfection. The cells were cotrans-fected with 0.1 *µ*g of either pMIR-ZEB2 or pMIR-REPORT, together with 20 nM miR-141 precursor molecule or 20 nM negative control #1 using siPORT amine transfection agent, according to the manufacturer's instructions. The pRL-TK vector (Promega Corp., Madison, WI, USA) containing *Renilla* luciferase was also cotransfected as a reference control. Firefly and *Renilla* luciferase activities were measured using Dual-Luciferase Reporter assay (Promega Corp.) 24 h post-transfection. The luciferase activity was measured using a luminometer (GloMax™ 96 Microplate Luminometer, Promega Corp.). Firefly luciferase activity was normalized to *Renilla* luciferase activity.

### Statistical analysis

The data are expressed as the mean ± standard error. Student's t-test was applied to analyze the differences between the groups, and statistical analysis was performed using SPSS software version 13 (SPSS, Inc., Chicago, IL, USA). P<0.05 was considered to indicate a statistically significant difference.

## Results

### Effects of miR-141 overexpression on the migration of gastric cancer cells

**Figure 1:**
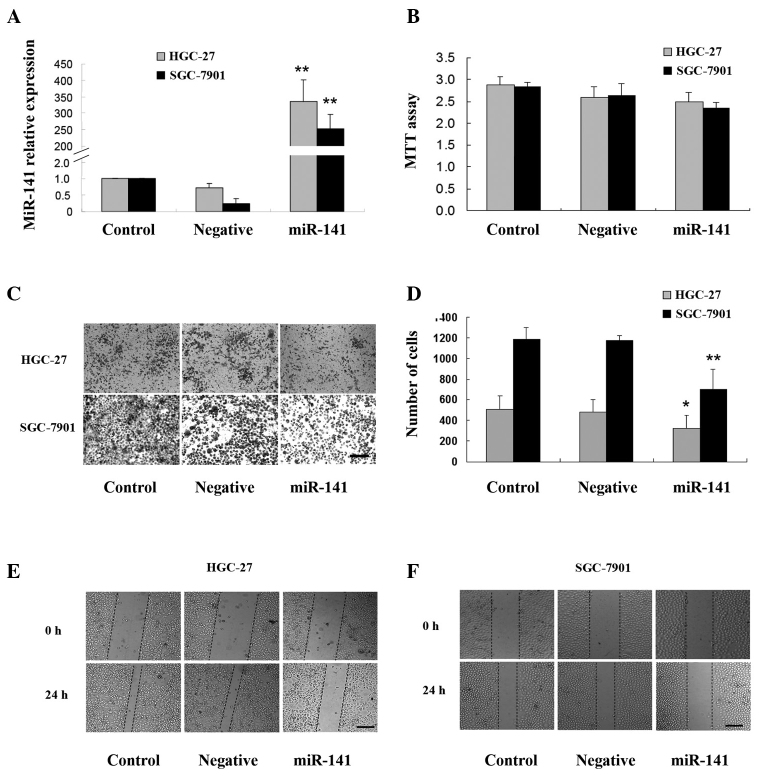
Effects of microRNA (miR)-141 overexpression on the migration of HGC-27 and SGC-7901 gastric cancer cells transfected with an miR-141 precursor (25 nM), a negative control (Negative, 25 nM), or neither of the above (Control) for 24 h were subjected to quantitative polymerase chain reaction (qPCR), MTT analysis, a cell migration assay, and a wound healing assay. (A) Relative expression levels of miR-141 in HGC-27 and SGC-7901 cells 24 h post-transfection were measured by qPCR. ^*^P<0.05, vs. Control. (B) Effects of miR-141 overexpression on the proliferation of HGC-27 and SGC-7901 cells were measured by MTT assay 24 h post-transfection. (C and D) Effects of miR-141 overexpression on the migration of HGC-27 and SGC-7901 cells were measured by cell migration assay 24 h post-transfection, and were (C) presented as phase contrast micrographs and (D) number of cells. (E and F) Effects of miR-141 overexpression on the migration of (E) HGC-27 and (F) SGC-7901 cells measured by wound healing assay 24 h post-transfection. Data are expressed as the mean ± standard error from three independent experiments. Scale bars represent 100*µ*m. ^*^P<0.05, ^**^P<0.01, vs. Negative. OD, optical density.

To explore the effects of miR-141 on the migration of gastric cancer cells, two human gastric mucous adenocarcinoma cell lines, HGC-27 and SGC-7901, were transfected with an miR-141 precursor or negative control precursor. The expression levels of miR-141 were upregulated by ~335 and 252 fold respectively in the HGC-27 and SGC-7901 cells transfected with miR-141 precursor, as compared with the control cells (Fig. 1A). The proliferation of both cell lines was not affected by miR-141 overexpression, as determined by MTT assay (Fig. 1B) over 24 h. The *in vitro* migration assay (Fig. 1C and D) and wound healing assay (Fig. 1E and F) demonstrated that overexpression of miR-141 markedly inhibited the migration of HGC-27 and SGC-7901 cells.

### ZEB2 is a target of miR-141

**Figure 2:**
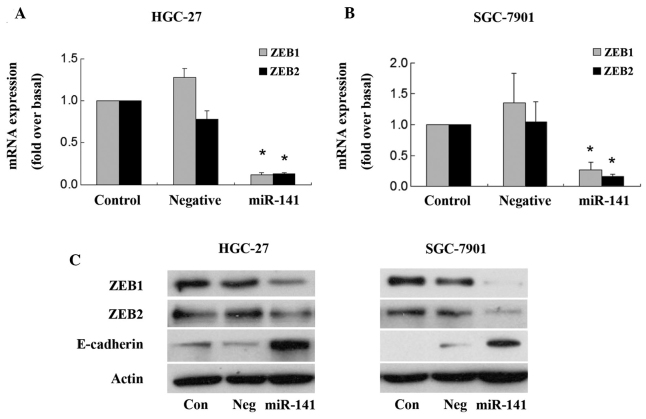
Effects of microRNA (miR)-141 on the expression levels of zinc finger E-box-binding homeobox (ZEB) in HGC-27 and SGC-7901 gastric cancer cells. The cells were transfected with miR-141 precursor (25 nM), negative control (Negative, 25 nM) or neither of the above (Control), and were subjected to quantitative polymerase chain reaction (qPCR) and western blotting. mRNA expression levels of ZEB1/2 were measured by qPCR in (A) HGC-27 and (B) SGC-7901 cells 24 h post-transfection. Data are expressed as the mean ± standard error from three independent experiments. ^*^P<0.05, vs. Negative. (C) Protein expression levels of ZEB1/2 and E-cadherin were detected by western blot analyses in HGC-27 and SGC-7901 cells. The expression of actin was used as a loading control.

To determine whether ZEBs are a target of miR-141 in gastric cancer, alterations in ZEB1/2 expression levels were determined post-transfection with an miR-141 precursor. As shown in Fig. 2, forced expression of miR-141 significantly reduced ZEB1/2 mRNA and protein expression levels in both HGC-27 and SGC-7901 gastric cancer cells. Conversely, overexpression of miR-141 upregulated the protein expression level of E-cadherin in both cell lines (Fig. 2C).

**Figure 3:**
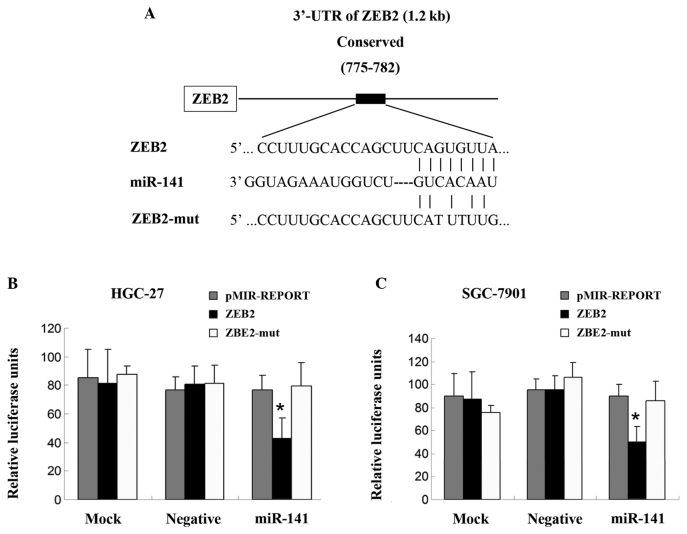
Zinc finger E-box-binding homeobox 2 (ZEB2) is the target of microRNA (miR)-141. (A) Schematic diagram of the putative miR-141-binding sites in the ZEB2 3′-untranslated region (UTR) region, as detected by TargetScan. ZEB2-mut indicates the ZEB2 3′-UTR with mutation in miR-141-binding sites. (B and C) Regulation of luciferase activity by ZEB2 3′-UTR is dependent on miR-141. (B) HGC-27 and (C) SGC-7901 human gastric cancer cells were transfected with the pRL-TK containing a *Renilla* luciferase gene, and the indicated vectors or precursors. Bars indicate the Firefly luciferase activities normalized to *Renilla* luciferase activities of the cotransfected pRL-TKvector. Each experiment was repeated at least three times, and each sample was assayed in triplicate. Data is expressed as the mean ± standard error from three independent experiments. ^*^P<0.05, vs. Negative.

To further confirm whether ZEB1/2 is directly regulated by miR-141, the 3′-UTR of ZEB2 was cloned with the predicted miR-141 binding sites downstream of a luciferase reporter gene (pMIR-ZEB2), and this vector was co-transfected with the miR-141 precursor or its negative control into HGC-27 and SGC-7901 cells. The luciferase activity of cells transfected with the miR-141 precursor was significantly decreased compared with the negative control. Furthermore, mutation of the putative miR-141-binding sites clearly eliminated the suppression of luciferase activity caused by miR-141 overexpression (Fig. 3A, B and C). These data suggest that miR-141 may inhibit ZEB2 protein expression through 3′-UTR at the posttranscriptional level.

### Expression of ZEB2 is inversely correlated with miR-141

**Figure 4:**
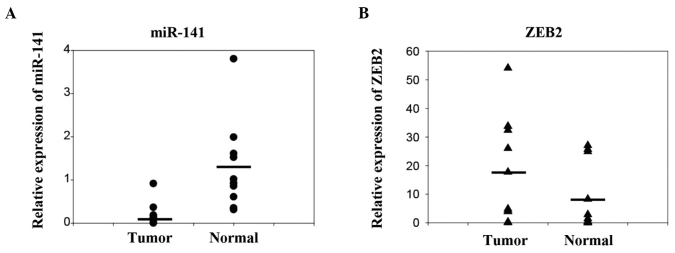
Zinc finger E-box-binding homeobox 2 (ZEB2) and microRNA (miR)-141 expression levels are inversely correlated in gastric cancer. (A) Expression levels of miR-141 and (B) ZEB2 mRNA in nine gastric tumor tissue samples and non-tumor tissue samples, as detected by quantitative polymerase chain reaction.

The mRNA expression levels of miR-141 and ZEB2 were detected in nine gastric tumor and non-tumor tissues by qPCR. The expression levels of miR-141 were significantly decreased in the gastric tumor tissue samples, compared with in the non-tumor tissue samples (Fig. 4A); however, mRNA expression levels of ZEB2 were increased in the gastric cancer tissue samples (Fig. 4B). The results suggest that the mRNA expression levels of ZEB2 may be inversely correlated with the expression levels of miR-141 in gastric tumor samples.

## Discussion

Numerous miRNAs have been reported to be involved in gastric cancer development (19). In our previous study, miR-141, which belongs to the miR-200 family, was shown to be downregulated in gastric cancer tissue samples and cell lines. In addition, upregulation of miR-141 significantly inhibited gastric cancer cell proliferation (8). The present study provided evidence suggesting that transfection with an miR-141 precursor suppresses the migration of gastric cancer cells. Overexpression of miR-141 significantly reduced the mRNA and protein expression levels of ZEB2, resulting in the upregulation of E-cadherin expression. By using a luciferase reporter assay, it was demonstrated that ZEB2 was the target of miR-141 in gastric cancer cell lines. Furthermore, the mRNA expression levels of ZEB2 mRNA were inversely correlated with the levels of miR-141 in gastric cancer tissue. The results of the present study suggest that miR-141 may have an important role in the inhibition and migration of gastric cancer cells, by targeting ZEB2.

miR-141 has previously been shown to function as a tumor suppressor in various cancers (9–11,16,20). It has been reported that low expression of miR-141 is a significant prognostic factor for poor overall survival, both in hepatocelluar carcinoma (HCC) (10) and pancreatic cancer (PC) (20). Upregulation of miR-141 resulted in a significant decrease in the migration and invasiveness of HCC and PC cancer cells (10,11,20). Similar findings have been obtained for colorectal cancer (CRC), as well as head and squamous cell carcinoma (9,16), indicating that miR-141 functions as an inhibitor of cancer metastasis by decreasing the migration and invasive potential of cancer cells. In our previous study, it was demonstrated that the expression levels of miR-141 in HGC-25 and SGC-7901 gastric cancer cell lines were lower as compared to that of MGC-803 poorly-differentiated human gastric mucous adenocarcinoma and BGC-823 undifferentiated human gastric cancer cell lines. Notably, both HGC-27 and SGC-7901 cells are derived from metastatic lymph nodes and are comparatively more invasive than the other two cell lines (8), which prompted us to evaluate the potential function of miR-141 in the metastasis of gastric cancer cells. The present study used HGC-27 and SGC-7901 cells as an *in vitro* model, and demonstrated that forced expression of miR-141 markedly inhibited the migration of gastric cancer cells.

The targets by which miR-141 regulates cancer cell motility varies in different types of cancer. miR-141 has been shown to inhibit the migration and invasion of HCC cells by targeting Tiam1 (10). In PC, both transmembrane 4 L six family member 1 and MAP4K4 have been reported to be directly targeted by miR-141 to regulate the invasion of PC cells (11,20). Chen *et al* (21) identified hepatoma-derived growth factor (HDGF) as a direct target of miR-141 in gastric cancer cells, and the suppressive effects of miR-141 on cancer cell migration and invasion were shown to be partially mediated by suppression of HDGF expression. The results of the present study demonstrated that ZEB2 was another direct target of miR-141.

ZEB2 is one of the transcriptional suppressors of E-cadherin, which is a key regulator of EMT. EMT constitutes an early and critical step during tumor invasion and metastasis, and loss of E-cadherin is considered a hallmark of EMT (22). During EMT, the expression of the miR-200 members, including miR-141, has been shown to be significantly down-regulated (14,23). Overexpression of miR-141 inhibits EMT by directly targeting the ZEBs and enhancing E-cadherin expression (14,23,24). In CRC cells, miR-141 was shown to regulate ZEB2, and inhibit the migration and invasion of CRC cells (9). In renal carcinoma cell lines, overexpression of miR-141 resulted in downregulation of ZEB2 and upregulation of E-cadherin (15). Recently, Wu *et al* (25) demonstrated that miR-141 functioned as a tumor suppressor via ZEB2 targeting HCC. Therefore, the present study hypothesized that in gastric cancer, downregulation of miR-141 may promote EMT, cancer cell migration, and invasion by targeting the E-cadherin transcriptional repressor ZEB2. However, further studies are required.

In conclusion, the present study demonstrated that overexpression of miR-141 inhibited gastric cancer cell migration. The miR-141 target ZEB2 was negatively regulated at both the transcriptional and post-transcriptional level by miR-141. These results suggest that miR-141 may suppress the metastasis of gastric cancer through its inhibitory effect on the EMT process. It may be interesting to further explore whether miR-141 could be used as a predictive biomarker for clinical outcomes, or as a therapeutic target to prevent gastric cancer progression.

## Figures and Tables

**Table I:** Patient information.

No.	Sample	Age	Gender	TNM	AJCC Stage	Differentiation	Recruitment Date
1	Tumor	61	M	T4aN3aM0	IIIC	Low	Oct 24, 2013
2	Tumor	60	M	T4aN3bM0	IIIC	Low	Nov 19, 2013
3	Tumor	71	F	T1bN0M0	IA	Low	Dec 1, 2013
4	Tumor	63	F	T4aN0M0	IIB	Low	Dec 1, 2013
5	Tumor	52	M	T4aN3bM0	IIIC	Low	Dec 24, 2013
6	Tumor	70	F	T4aN3aM0	IIIC	Moderate	Dec 25, 2013
7	Tumor	83	F	T4aN0M0	IIB	Low	Dec 25, 2013
8	Tumor	64	F	T4aNxM1	IV	Low	Dec 25, 2013
9	Tumor	70	F	T4aN0M0	IIB	Low	Jan 9, 2014
10	Non-tumor	66	M	None	None	None	Oct 23, 2013
11	Non-tumor	66	F	None	None	None	Oct 23, 2013
12	Non-tumor	51	F	None	None	None	Oct 24, 2013
13	Non-tumor	53	F	None	None	None	Oct 31, 2013
14	Non-tumor	45	M	None	None	None	Oct 31, 2013
15	Non-tumor	41	F	None	None	None	Nov 12, 2013
16	Non-tumor	38	F	None	None	None	Dec 13, 2013
17	Non-tumor	30	M	None	None	None	Dec 24, 2013
18	Non-tumor	48	F	None	None	None	Dec 24, 2013

TNM and Cancer Stage are defined according to the 7th edition of the AJCC cancer staging manual published in 2010. TNM, tumor, lymph node, metastasis; F, female; M, male; AJCC, American Joint Committee on Cancer.
